# Military training elicits marked increases in plasma metabolomic signatures of energy metabolism, lipolysis, fatty acid oxidation, and ketogenesis

**DOI:** 10.14814/phy2.13407

**Published:** 2017-09-12

**Authors:** J. Philip Karl, Lee M. Margolis, Nancy E. Murphy, Christopher T. Carrigan, John W. Castellani, Elisabeth H. Madslien, Hilde‐Kristin Teien, Svein Martini, Scott J. Montain, Stefan M. Pasiakos

**Affiliations:** ^1^ Military Nutrition Division U.S. Army Research Institute of Environmental Medicine Natick Massachusetts; ^2^ Oak Ridge Institute for Science and Education Oak Ridge Tennessee; ^3^ Thermal and Mountain Medicine Division U.S. Army Research Institute of Environmental Medicine Natick Massachusetts; ^4^ Norwegian Defense Research Establishment Kjeller Norway

**Keywords:** Endurance exercise, energy deficit, metabolism, metabonomics, physiology

## Abstract

Military training studies provide unique insight into metabolic responses to extreme physiologic stress induced by multiple stressor environments, and the impacts of nutrition in mediating these responses. Advances in metabolomics have provided new approaches for extending current understanding of factors modulating dynamic metabolic responses in these environments. In this study, whole‐body metabolic responses to strenuous military training were explored in relation to energy balance and macronutrient intake by performing nontargeted global metabolite profiling on plasma collected from 25 male soldiers before and after completing a 4‐day, 51‐km cross‐country ski march that produced high total daily energy expenditures (25.4 MJ/day [SD 2.3]) and severe energy deficits (13.6 MJ/day [SD 2.5]). Of 737 identified metabolites, 478 changed during the training. Increases in 88% of the free fatty acids and 91% of the acylcarnitines, and decreases in 88% of the mono‐ and diacylglycerols detected within lipid metabolism pathways were observed. Smaller increases in 75% of the tricarboxylic acid cycle intermediates, and 50% of the branched‐chain amino acid metabolites detected were also observed. Changes in multiple metabolites related to lipid metabolism were correlated with body mass loss and energy balance, but not with energy and macronutrient intakes or energy expenditure. These findings are consistent with an increase in energy metabolism, lipolysis, fatty acid oxidation, ketogenesis, and branched‐chain amino acid catabolism during strenuous military training. The magnitude of the energy deficit induced by undereating relative to high energy expenditure, rather than macronutrient intake, appeared to drive these changes, particularly within lipid metabolism pathways.

## Introduction

Military operations commonly require prolonged physical exertion in austere environments resulting in high energy expenditures that are generally not matched by increases in energy intake (Friedl et al. 1994, 2000; Tharion et al. 2005; Margolis et al. 2013, 2014). This provides opportunities for studying the range of human physiologic responses to severe energy deficit in combination with multiple additional stressors (e.g., sleep deprivation, psychological stress). Previous investigations have demonstrated that arduous military trainings result in an increased mobilization of fatty acids and amino acids to support ketogenesis and gluconeogenesis, respectively, concurrent to a transition into a catabolic and proinflammatory state (Friedl et al. 2000; Nindl et al. 2003; Li et al. 2013; McClung et al. 2013; Margolis et al. 2014; Karl et al. 2016; Pasiakos et al. 2016; Berryman et al. 2017), and that nutrition during training and recovery may modulate these effects (Nindl et al. 1997; Alemany et al. 2008; Pikosky et al. 2008; Pasiakos et al. 2013; Margolis et al. 2016) and ultimately impact performance (Welsh et al. 2008; Fortes et al. 2011; Margolis et al. 2014). However, monitoring metabolic status, identifying molecules and metabolic pathways underpinning decrements in performance in these environments, and assessing the effectiveness of nutritional strategies for mitigating these decrements have historically relied on measuring a relatively small number of select biomarkers. An alternative approach utilizes nontargeted metabolomics to capture whole‐body metabolic responses by simultaneously measuring hundreds to thousands of small molecules (<1500 Da; metabolites) in a small volume of biological sample (Nicholson et al. 1999). This approach can provide more comprehensive and sensitive markers of metabolic status than commonly measured biomarkers while offering insights into the regulation of metabolic pathways (Sampson et al. 2014). Metabolomics may therefore provide an efficient method of assessing dynamic metabolic responses to stress and nutritional interventions during military training, and for identifying novel mechanisms modulating performance in these environments (Bradburne et al. 2015; DelRaso et al. 2015).

In support, Phua et al. (2015) recently demonstrated that military combat training in hot and humid conditions produced a distinct urine metabolite profile in Singaporean soldiers, and identified several novel relationships between metabolites and performance‐related health outcomes. However, to our knowledge, no other study has utilized metabolomics for characterizing physiologic responses to military training. Recent metabolomics studies conducted in civilian cohorts have demonstrated that single and repeated endurance exercise bouts elicit rapid changes in whole‐body substrate utilization characterized by an integrated multiorgan response that simultaneously upregulates lipolysis, fatty acid oxidation, amino acid oxidation, ketogenesis, and glycolysis, and which partially persists for 14–21 h following cessation of exercise (Lewis et al. 2010; Nieman et al. 2013b, 2014). This postexercise metabolomic profile demonstrates strong similarity to that experienced during short‐term fasting as measured by both metabolomics (Rubio‐Aliaga et al. 2011; Krug et al. 2012) and more conventional approaches (Cahill 2006), and can be influenced by dietary intake (Chorell et al. 2009; Krug et al. 2012; Nieman et al. 2013a). Collectively these studies have used metabolomics to link dietary intake, catabolism, anabolism, and oxidative stress during exercise and in the postexercise recovery period (Lehmann et al. 2010; Krug et al. 2012; Nieman et al. 2013b, 2014), and identified novel potential biomarkers of physical activity (Lehmann et al. 2010; Lewis et al. 2010), oxidative stress (Nieman et al. 2013b, 2014), and dietary intake (Claus and Swann 2013; Kinross et al. 2014). However, these studies have generally focused on single physiologic stressors and have not examined whole‐body metabolic responses to the combination of prolonged physical activity and undernutrition that is characteristic of military training. In the present study, whole‐body metabolic responses to strenuous military training were explored in relation to energy balance and macronutrient intake by performing nontargeted global metabolite profiling on plasma collected from soldiers before and after completing a short‐term (4‐day) military training exercise that elicited a severe energy deficit (13.8 MJ/day), body mass loss, and negative nitrogen balance (Margolis et al. 2016).

## Materials and Methods

### Participants

Data for this investigation were collected on 25 of 73 male Norwegian Army Soldiers (19 years [SD 1], BMI 23 kg/m^2^ [SD 2]) participating in a randomized controlled trial assessing the impact of protein or carbohydrate supplementation on energy balance and whole‐body protein retention during a 4‐day arctic military training exercise. Primary study outcomes have been previously reported (Margolis et al. 2016; Pasiakos et al. 2016; Karl et al. 2017), and the present analysis reports secondary study outcomes. All participants were soldiers stationed in Skjold, Norway, and provided informed written consent before being randomized to one of three dietary treatment groups, each receiving three Norwegian arctic combat rations, either alone (control; CNTRL) or supplemented with whey protein‐based (PRO) or carbohydrate (maltodextrin)‐based (CHO) snack bars daily. The study was approved by the Institutional Review Board at the U.S. Army Research Institute of Environmental Medicine (Natick, MA) and the Regional Committees for Medical and Health Research Ethics (REK sør‐øst, Oslo, NO). Investigators adhered to the policies for protection of human subjects as prescribed in 32 CFR Part 219, U.S. Department of Defense Instruction 3216.02 (Protection of Human Subjects and Adherence to Ethical Standards in DoD‐Supported Research) and Army Regulation 70‐25. The trial was registered on www.clinicaltrials.gov as NCT02327208.

### Experimental design

Before initiating the training exercise (PRE; study day −1 and 0), height and body mass were measured, and a fasting blood sample was collected. Beginning on day 1, participants began a 51‐km cross‐country ski march carrying an approximately 45 kg pack. Participants skied in 50:10 min work to rest ratios, traveling a total distance of approximately 13 km/day for 4 days. Energy expenditure and energy and macronutrient intakes were assessed daily. Body mass was measured and a fasted blood sample was collected after finishing the course (POST; study day 5). The timing of POST depended on when each participant finished the course. Participants finishing earlier (*n *=* *17) did so in the late evening of study day 4, and were able to sleep for a few hours prior to an early morning blood draw 8–10 h after completing the course. Participants finishing midmorning on study day 5 (*n *=* *7) were tested 2–3 h after completing the course. All participants reported complying with instructions to fast overnight prior to POST.

### Dietary intake and energy balance

On study day −1, participants were provided three Norwegian arctic combat rations to consume daily throughout the study. At initiation of training (study day 1), participants assigned to PRO and CHO were also provided with four snack bars per day to be consumed in addition to the provided rations. Participants were asked to consume the rations and bars as they normally would during training, and no outside food was permitted. Energy and macronutrient intakes were calculated from food logs collected from participants and reviewed by staff daily. Nutritional composition of all combat ration items included in this study was confirmed by chemical analysis (Covance Laboratories, Inc., Madison, WI). Investigators, study staff, and participants were blind to the macronutrient composition of the bars.

Energy expenditure was assessed using doubly labeled water as previously described (Margolis et al. 2016). Briefly, fasted participants provided a baseline urine sample, and then ingested the doubly labeled water (H_2_^18^O and ^2^H_2_O; Sigma‐Aldrich, St. Louis, MO). Urine samples were collected at 4 and 6 h after dosing, and daily thereafter to determine peak enrichment and isotopic elimination rates as previously described (Margolis et al. 2016). Determination of CO_2_ production was performed according to Schoeller et al. (1986), and used to calculate energy expenditure with the energy equivalent of CO_2_ for a respiratory quotient of 0.86 based on the average food quotient for the course.

### Plasma metabolomics

Blood was collected following an overnight fast (>8 h) by antecubital venipuncture into collection tubes containing K2‐EDTA, separated into plasma, and immediately frozen. Samples were then shipped on dry ice to the U.S. Army Research Institute of Environmental Medicine where they were stored until being shipped to Metabolon, Inc. (Durham, NC) for analysis.

Metabolomics analysis proceeded as previously described (Karl et al. 2017). Briefly, samples were analyzed using four separate methods: two separate reverse‐phase (RP)/UPLC‐MS/MS methods with positive ion mode electrospray ionization (ESI), a RP/UPLC‐MS/MS method with negative ion mode ESI, and a HILIC/UPLC‐MS/MS method with negative ion mode ESI. All analysis methods utilized a Waters ACQUITY UPLC (Waters Corp., Milford, MA) and a Thermo Scientific Q‐Exactive high‐resolution/accurate mass spectrometer interfaced with a heated ESI‐II source and Orbitrap mass analyzer operated at 35,000 mass resolution. Technical replicates, blanks, internal standards, and several recovery standards were analyzed with experimental samples for quality control. Raw data were extracted, peak identified, and quality control processed using proprietary hardware and software (Metabolon, Inc.). Identification of peaks was based on comparing retention times, mass to charge ratios, and chromatographic data retained within a library maintained by Metabolon which contains entries of purified standards and recurrent unknown entities. Peaks were quantified using area under the curve which was used in statistical analyses.

### Statistical analysis

Sample size calculations were based on primary study outcomes which have been previously reported (Margolis et al. 2016; Pasiakos et al. 2016). Analyses were completed using R v.3.3.1, SPSS v.21 (IBM Analytics; Armonk, NY), ArrayStudio (Omicsoft Corp.; Cary, NC), and MetaboAnalyst v.3.0 (Xia and Wishart 2016). Data were examined for normality prior to analysis and transformed if necessary to meet model assumptions. Prior to analysis of plasma metabolites, any missing values were imputed using the minimum observed value for each compound, normalized to set the median equal to 1, and log_10_ transformed.

Energy expenditure and dietary intake were compared across groups by one‐way ANOVA. Plasma metabolite profiles were analyzed using principal components analysis, hierarchical complete‐linkage clustering of Euclidean distances, and random forest analysis to determine whether global metabolite profiles differentiated individuals based on time point or diet group. The effects of training and diet, and their interaction on individual plasma metabolites were examined by repeated measures ANOVA.

Exploratory analyses of associations between metabolites related to energy metabolism (lipid, amino acid, energy, and nucleotide super pathways) that significantly changed from PRE to POST (*Q *<* *0.10) and measures of energy balance and dietary intake were also conducted. These analyses were prompted by the small sample sizes within diet groups, overlaps in energy and macronutrient intake between groups, an apparent impact of training on multiple energy metabolism pathways (see Results), and the known independent effects of dietary intake on energy metabolism pathways. Linear associations between log_10_‐transformed changes in metabolite levels with changes in body weight, and energy balance, energy expenditure, and energy and macronutrient intakes during training were assessed by Spearman's correlation. To assess the association between changes in metabolite levels and macronutrient intake independent of total energy intake, macronutrient intakes were adjusted for total energy intake using the residual method (Willett 1998) prior to correlation analyses.

One individual who did not provide a POST blood sample was excluded from repeated measures and correlational analyses that included metabolomics data. The false discovery rate for all tests that included metabolite data was controlled by adjusting *P* values using the Benjamini–Hochberg procedure. Adjusted *P* values are presented as *Q* values. Data are presented as mean (SD) unless otherwise noted. Statistical significance was set at *P *≤* *0.05 or *Q *≤* *0.20.

## Results

The 25 individuals included in this report were a self‐selected cohort who chose to participate in an optional study procedure, results of which have been reported elsewhere (Karl et al. 2017). This subgroup did not differ in age (*P *=* *0.59), BMI (*P *=* *0.47), body mass loss (*P *=* *0.98), energy intake (*P *=* *0.51), macronutrient intake (*P *≥* *0.11), or energy expenditure (*P *=* *0.94) relative to the other participants. Weight loss averaged 2.8 kg (SD 1.0), and did not differ by diet group (*P *=* *0.51). Fourteen of the 25 individuals participated in the doubly labeled water measurement, and demonstrated high energy expenditures of 25.4 MJ/day (SD 2.3; range: 22.0 MJ/day, 29.6 MJ/day), and severe energy deficits of 13.6 MJ/day (SD 2.5; range: 10.6 MJ/day, 19.5 MJ/day), neither of which differed by diet group (*P *≥* *0.67). Macronutrient intakes varied across study groups as planned with higher mean protein intakes in PRO relative to CNTRL and CHO, and higher mean carbohydrate intake in CHO relative to PRO (Table**** 1).

**Table 1:** Energy and macronutrient intakes during a 4‐day, 51‐km cross‐country ski march

	CNTRL(*n *=* *5)	CHO(*n *=* *9)	PRO(*n *=* *10)	*P*‐value
Energy intake, MJ/day	11.5 (1.7) [9.5, 13.0]	13.4 (2.7) [7.8, 16.3]	12.4 (2.5) [8.6, 16.8]	0.38
Protein intake, g/day	109 (14) [92, 124]	100 (21) [66, 123]	155 (26)^a^[108, 191]	<0.001
Carbohydrate intake, g/day	340 (51) [281, 285]	443 (82)^b^ [252, 513]	335 (82) [189, 490]	0.01
Fat intake, g/day	102 (17) [83, 117]	111 (27) [62, 146]	109 (22) [70, 141]	0.76

Values are mean (SD) and range [min, max]. One individual excluded from PRO due to incomplete food logs. CNTRL, control group; CHO, carbohydrate supplement group; PRO, protein supplement group. Values compared by one‐way ANOVA with Tukey's correction.

a Significantly different from CNTRL and CHO, *P *<* *0.01.

b Significantly different from PRO, *P *=* *0.02.

### Plasma metabolites

**Figure 1:**
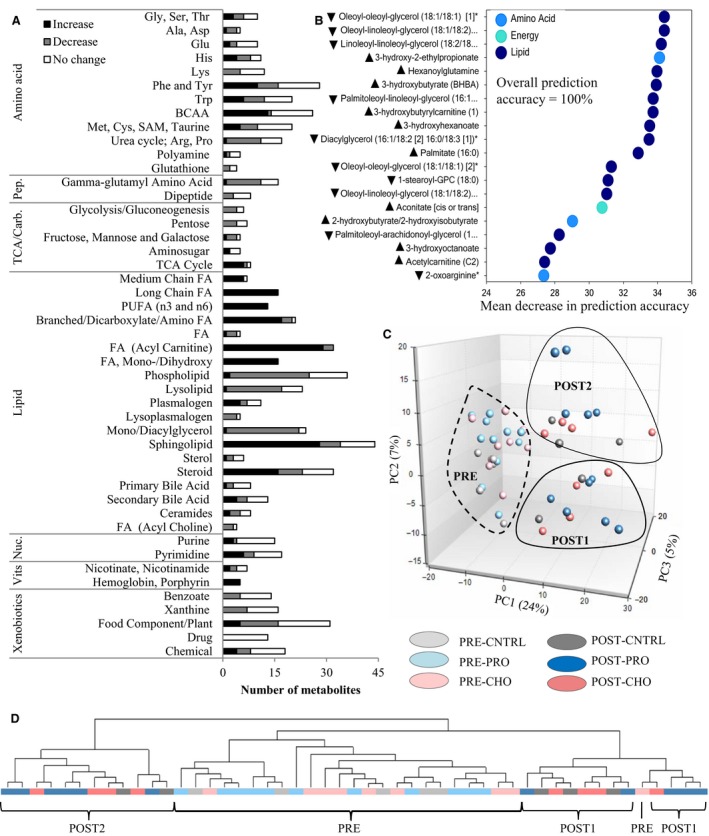
Military training elicits changes in plasma metabolite profiles. (A) Number of metabolites within each subpathway that significantly increased (black fill), decreased (gray fill), or did not change (white fill) from pre‐ to post‐training (*n *=* *24; repeated measures ANOVA main effect of time, *Q *<* *0.10). Only pathways with ≥2 metabolites are shown. (B) Top 20 metabolites with the strongest influence on the prediction accuracy of the random forest analysis are presented in order of importance (top to bottom). Random forest analysis used individual metabolite profiles to predict whether the samples were collected pre‐ or post‐training (*n *=* *25). Mean decrease in prediction accuracy is the mean decrease in the percentage of observations classified correctly when that metabolite is assigned a random value. Larger mean decrease accuracy indicates greater importance to the overall prediction accuracy of the analysis. Arrows indicate direction of metabolite change from PRE to POST (main effect of time, *Q *<* *0.10). (C) Principal components (PC) plot of plasma metabolite profiles *(n *=* *25). Individual data points represent the metabolite composition within a single individual. Points closer together are more similar. PC1, PC2, and PC3, respectively, account for 24%, 7%, and 5% of the variability in plasma metabolite profiles. (D) Hierarchical complete‐linkage clustering of Euclidean distances of plasma metabolite profiles (*n *=* *25). Branches (lines) within the same node (points where branches split) reflect similarity in metabolite composition. CHO, carbohydrate supplement group; CNTRL, control group (rations only); PRO, protein supplement group; BCAA, branched‐chain amino acid; Carb., carbohydrate; FA, fatty acid; Nuc., nucleotide; Pep., peptide; PUFA, polyunsaturated FA; TCA, tricarboxylic acid; SAM,* S*‐adenosylmethionine; Vits, vitamins.

Concentrations of 478 of the 737 identified metabolites changed from PRE to POST training (main effect of time, *Q < *0.10) (Fig. 1A and Table S1). Principal components (Fig. 1C) and hierarchical cluster (Fig. 1D) analyses of plasma compounds demonstrated a clear separation of samples by time point but not by diet. These analyses further separated POST samples into two distinct clusters of 12 participants independent of diet group (Fig. 1C and D). Within these two clusters, all participants who had their blood drawn within 2–3 h of completing the course (i.e., late completers, *n *=* *7) were assigned to the same cluster (POST2 in Fig. 1C and D). Mean weight loss was 0.9 kg [95% CI: 0.2, 1.7 kg] greater in POST2 relative to POST1, but energy expenditure (*P *=* *0.44), energy intake (*P *=* *0.18), energy deficit (*P *=* *0.22), and macronutrient intakes (*P *≥* *0.19) did not differ between clusters.

Random forest analyses correctly differentiated PRE and POST plasma samples with 100% accuracy (Fig. 1B) while correctly differentiating samples by diet group with only 25% accuracy. Sixteen of the top 20 metabolites with the greatest influence on discriminating PRE from POST samples in the random forest analysis were related to lipid metabolism, 3 to amino acid metabolism, and 1 to TCA cycle metabolism (Fig. 1B).

The most consistent and largest changes in metabolite levels from PRE to POST were seen in subpathways related to lipolysis and fatty acid oxidation (Fig.**** 1A and Table S1). Decreases from PRE to POST (main effect of time, *Q *<* *0.10) were observed for 88% of the detected mono‐ and diacylglycerols, 63% of the phospholipids, and 70% of the lysolipids, many of which decreased by >50% (Table**** 2). In contrast, the majority of detected free fatty acids (FFA) increased (main effect of time, *Q *<* *0.10) (Fig.**** 1A). This included increases in 100% of the long‐chain and polyunsaturated fatty acids, 86% of the medium‐chain fatty acids, 81% of the branched‐chain and dicarboxylate fatty acids, and 100% of the monohydroxy fatty acids, many of which were >twofold higher POST relative to PRE (Table 3). Concurrent increases in 91% of the measured acylcarnitines and >20‐fold increases in the ketone bodies 3‐hydroxybutyrate and acetoacetate were also observed (Table 4).

**Table 2:** Plasma metabolites decreasing by >50% and related to diacylglycerol, phospholipid, and lysolipid metabolism measured before and after a 4‐day, 51‐km cross‐country ski march

Subpathway	Biochemical name	Fold change (POST/PRE)
Diacylglycerol	**Diacylglycerol (16:1/18:2 [2], 16:0/18:3 [1])** b	**0.19**
	Oleoyl‐linoleoyl‐glycerol (18:1/18:2) [1]	0.31
	**Oleoyl‐linoleoyl‐glycerol (18:1/18:2) [2]**	**0.29**
	**Linoleoyl‐linoleoyl‐glycerol (18:2/18:2) [1]** b	**0.30**
	Linoleoyl‐linolenoyl‐glycerol (18:2/18:3) [1]^b^	0.21
	**Palmitoleoyl‐linoleoyl‐glycerol (16:1/18:2)[1]** a ^**,**^ b	**0.19**
	Palmitoyl‐oleoyl‐glycerol (16:0/18:1) [1]^b^	0.26
	Palmitoyl‐oleoyl‐glycerol (16:0/18:1) [2]^b^	0.27
	Palmitoyl‐linoleoyl‐glycerol (16:0/18:2) [1]^b^	0.18
	Palmitoyl‐linoleoyl‐glycerol (16:0/18:2) [2]^b^	0.31
	Oleoyl‐oleoyl‐glycerol (18:1/18:1) [1]^b^	**0.28**
	**Oleoyl‐oleoyl‐glycerol (18:1/18:1) [2]** b	**0.25**
	**Palmitoleoyl‐arachidonoyl‐glycerol (16:1/20:4) [2]** a ^**,**^ b	**0.12**
Lysolipid	**1‐Stearoyl‐GPC (18:0)** a	**0.47**
	1‐Linolenoyl‐GPE (18:3)^b^	0.49
Phospholipid	1‐Linoleoyl‐2‐linolenoyl‐GPC (18:2/18:3)^b^	0.49
	1,2‐Dilinoleoyl‐GPE (18:2/18:2)^b^	0.39

Data are fold changes calculated using the mean for each time point. Repeated measures ANOVA (*n *=* *24) used to examine main effect of time, diet, and their interaction on metabolites measure before (PRE) and after (POST) military training. *P* values were adjusted using the Benjamini–Hochberg correction (*Q*); main effect of time, *Q *<* *0.10 for all. All diet by time interactions were not statistically significant (*Q *>* *0.20). Metabolites in bold font are those with the strongest influence on prediction accuracy in the random forest analysis (see Fig. 1B).

a Metabolite was >50% lower in the cluster containing participants who finished the course late (POST2 vs. POST1, independent samples *t* test; *Q *<* *0.10).

b Compounds that have not been officially confirmed based on a standard, but are identified with high confidence.

**Table 3:** Plasma fatty acids demonstrating >twofold changes before and after a 4‐day, 51‐km cross‐country ski march

Subpathway	Biochemical name	Fold change (POST/PRE)
Medium‐chain fatty acid	Caprate (10:0)	2.07
	10‐Undecenoate (11:1n1)	2.10
	Laurate (12:0)	3.32
	5‐Dodecenoate (12:1n7)	5.36
Long‐chain fatty acid	Myristate (14:0)	4.12
	Myristoleate (14:1n5)	5.70
	Pentadecanoate (15:0)	2.04
	**Palmitate (16:0)**	**2.20**
	Palmitoleate (16:1n7)	4.74
	Margarate (17:0)	2.73
	10‐Heptadecenoate (17:1n7)	4.24
	Nonadecanoate (19:0)	2.07
	10‐Nonadecenoate (19:1n9)	4.34
	Arachidate (20:0)	2.08
	Eicosenoate (20:1)	4.71
	Erucate (22:1n9)	3.42
	Oleate/vaccenate (18:1)	2.51
Polyunsaturated fatty acid (n3 and n6)	Stearidonate (18:4n3)	3.16
	Docosapentaenoate (n3 DPA; 22:5n3)	2.73
	Linoleate (18:2n6)	2.86
	Linolenate [alpha or gamma; (18:3n3 or 6)]	4.06
	Adrenate (22:4n6)	2.39
	Docosadienoate (22:2n6)	3.12
	Dihomolinoleate (20:2n6)	3.93
Fatty acid, branched	13‐Methylmyristate	2.22
	15‐Methylpalmitate	2.88
	17‐methylstearate	2.49
Fatty acid, dicarboxylate	Adipate	4.50
	3‐Methyladipate	3.59
	Sebacate (decanedioate)^a^	6.06
	Dodecanedioate	4.40
	Tetradecanedioate	3.59
	Hexadecanedioate	3.08
	Octadecanedioate	2.82
Fatty acid, monohydroxy	**3‐Hydroxyhexanoate** a	**6.25**
	**3‐Hydroxyoctanoate** a	**5.02**
	3‐Hydroxydecanoate^a^	4.68
	3‐Hydroxysebacate	10.04
	3‐Hydroxylaurate	3.71
	5‐Hydroxyhexanoate	2.68
	13‐HODE + 9‐HODE	2.45
	9‐Hydroxystearate	3.09
**Fatty acid, acyl glutamine**	**Hexanoylglutamine**	**13.80**

Data are fold changes calculated using the mean for each time point. Repeated measures ANOVA (*n *=* *24) used to examine main effect of time, diet, and their interaction on metabolites measure before (PRE) and after (POST) military training. *P* values were adjusted using the Benjamini–Hochberg correction (*Q*); main effect of time, *Q *<* *0.10 for all. All diet by time interactions were not statistically significant (*Q *>* *0.20). Metabolites in bold font are those with the strongest influence on prediction accuracy in the random forest analysis (see Fig. 1B). HODE, hydroxyl‐octadecadienoic acid.

a Metabolite was >twofold higher in the cluster containing participants who finished the course late (POST2 vs. POST1, independent samples *t* test; *Q *<* *0.10).

**Table 4:** Metabolites demonstrating >twofold changes and related to acylcarnitine and ketone body metabolism measured before and after a 4‐day, 51‐km cross‐country ski march

Subpathway	Biochemical name	Fold change (POST/PRE)
Acylcarnitine	Hexanoylglycine	2.54
	**Acetylcarnitine (C2)**	**2.43**
	**3‐Hydroxybutyrylcarnitine (1)**	**25.05**
	3‐Hydroxybutyrylcarnitine (2)	4.01
	Hexanoylcarnitine (C6)	2.76
	Octanoylcarnitine (C8)	2.46
	Decanoylcarnitine (C10)	2.56
	*Cis*‐4‐decenoylcarnitine (C10:1)	2.20
	Laurylcarnitine (C12)	3.28
	Myristoylcarnitine (C14)	2.60
	Palmitoleoylcarnitine (C16:1)^a^	3.06
	Myristoleoylcarnitine (C14:1)^a^	4.40
	Suberoylcarnitine (C8‐DC)	7.61
	Adipoylcarnitine (C6‐DC)	6.32
	Arachidoylcarnitine (C20)^a^	2.49
	Erucoylcarnitine (C22:1)^a^	3.94
Ketone bodies	Acetoacetate	20.59
	**3‐Hydroxybutyrate**	**31.25**

Data are fold changes calculated using the mean for each time point. Repeated measures ANOVA (*n *=* *24) used to examine main effect of time, diet, and their interaction on metabolites measure before (PRE) and after (POST) military training. *P* values were adjusted using the Benjamini–Hochberg correction (*Q*); main effect of time, *Q *<* *0.10 for all. All diet by time interactions were not statistically significant (*Q *>* *0.20). Metabolites in bold font are those with the strongest influence on prediction accuracy in the random forest analysis (see Fig. 1B).

a Compounds that have not been officially confirmed based on a standard, but are identified with high confidence.

Amino acid metabolism was also affected by the training (Fig. 1A, Table 5, and Table S1). Half of the metabolites related to branched‐chain amino acid (BCAA) metabolism increased from PRE to POST, although fold changes were generally <2 (main effect of time, *Q *<* *0.10). In contrast, decreased levels of 59% of metabolites related to urea cycle activity were observed. The largest fold changes in amino acid metabolites, however, were observed for those related to histidine, methionine, cysteine, *S*‐adenosylmethionine, and taurine metabolism (Table 5).

**Table 5:** Metabolites demonstrating >50% decreases or >twofold changes related to amino acid metabolism measured before and after a 4‐day, 51‐km cross‐country ski march

Subpathway	Biochemical name	Fold change (POST/PRE)
Histidine	3‐Methylhistidine	0.34
	*N*‐Acetyl‐3‐methylhistidine^a^	0.32
Phenylalanine and tyrosine	Gentisate	0.42
	Vanillic alcohol sulfate	2.29
Tryptophan	Indoleacetylglutamine	0.47
Branched‐chain amino acids	**3‐Hydroxy‐2‐ethylpropionate**	**2.63**
	Ethylmalonate	2.07
Methionine, cysteine, *S*‐adenosylmethionine, and taurine	*S*‐Methylmethionine	0.35
	Alpha‐ketobutyrate	3.14
	*N*‐Methyltaurine	0.36
	**2‐Hydroxybutyrate/2‐hydroxyisobutyrate**	**3.23**
Urea cycle; arginine and proline metabolism	**2‐Oxoarginine** a	**0.43**

Data are fold changes calculated using the mean for each time point. Repeated measures ANOVA (*n *=* *24) used to examine main effect of time, diet, and their interaction on metabolites measure before (PRE) and after (POST) military training. *P* values were adjusted using the Benjamini–Hochberg correction (*Q*); main effect of time, *Q *<* *0.10 for all. All diet by time interactions were not statistically significant (*Q *>* *0.20). Metabolites in bold font are those with the strongest influence on prediction accuracy in the random forest analysis (see Fig. 1B).

a Compounds that have not been officially confirmed based on a standard, but are identified with high confidence.

Several metabolites related to TCA cycle activity increased from PRE to POST (main effect of time, *Q *<* *0.10) (Fig. 1A and Table S1). This included the TCA cycle intermediates citrate, *cis*‐aconitate, *α*‐ketoglutarate, fumarate, and malate, which all increased 1.1‐ to 1.9‐fold. In contrast, 67% of measured metabolites related to glycolysis and gluconeogenesis to include glucose, pyruvate, lactate, and glycerate decreased from PRE to POST (main effect of time, *Q *<* *0.10).

Metabolism of steroid hormones was also affected by the training (Fig. 1A and Table S1). Half of the metabolites related to steroid metabolism increased from PRE to POST, all by <twofold, (main effect of time, *Q *<* *0.10), including several related to progesterone and corticosterone metabolism (e.g., 21‐hydroxypregnenolone disulfate, 17‐*α*‐hydroxypregnenolone sulfate, 5‐*α*‐pregnan‐3‐*β*, 20‐*α*‐diol disulfate, and pregnen‐diol disulfate), and several related to androgen synthesis (e.g., dehydroisoandrosterone sulfate, 4‐androsten‐3*α*, 17*α*‐diol monosulfate, 4‐androsten‐3*β*,17*β*‐diol disulfate, and andro steroid monosulfate).

Twenty‐six metabolites demonstrated ≥twofold differences between the two clusters identified in the POST samples. Nine of the compounds were related to caffeine metabolism, all of which were lower in the POST2 cluster. Other metabolites were largely consistent with the PRE versus POST comparisons. Specifically, two diacylglycerol metabolites were lower and four fatty acid metabolites were higher in POST2 relative to POST1. Other metabolites were assigned to multiple different subpathways, and only five of the 26 metabolites were among those demonstrating the largest fold changes from PRE to POST (Tables 2, 3, 4, 5). These observations suggested that the timing of sample collection relative to course completion likely had minimal impact on the most robust changes seen from pre‐ to post‐training.

### Associations between metabolites, energy balance, and dietary intake

**Figure 2:**
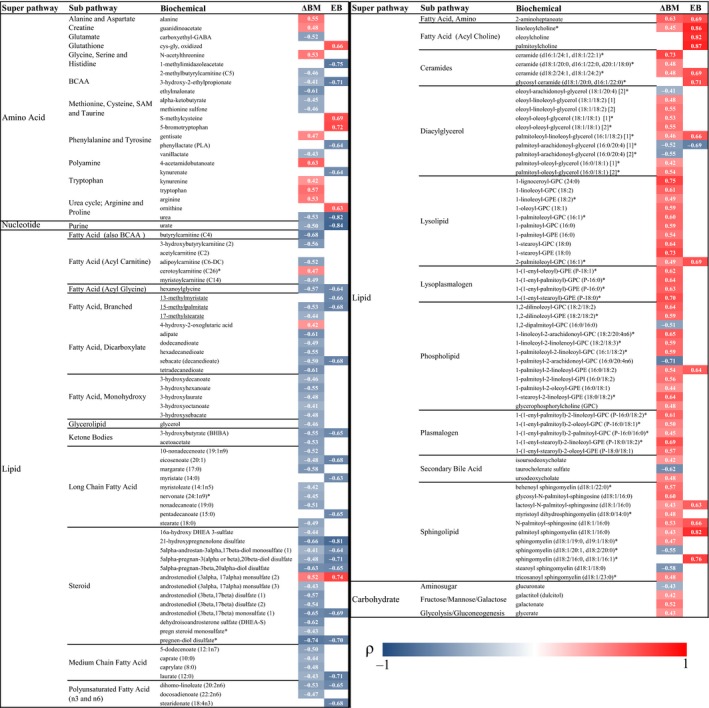
Body weight loss and energy balance correlate with changes in metabolite levels. Log_10_‐transformed changes in metabolite levels were correlated with changes in body mass (ΔBM,* n *=* *24) and measured energy balance (EB,* n *=* *13) during military training using Spearman's correlation. Inverse associations indicate that a decrease in body mass or more negative EB was associated with a larger fold change in plasma metabolite levels. *P*‐values adjusted using the Benjamini–Hochberg correction (*Q*). Values within cells are correlation coefficients (*ρ*). Only statistically significant correlations are presented (*Q *<* *0.20).

Body mass loss was correlated with changes in the levels of 128 metabolites (*Q *<* *0.20; Figure 2). Several noticeable patterns were observed including multiple inverse correlations between body mass loss and metabolites related to dicarboxylate fatty acid, monohydroxy fatty acid, long‐chain fatty acid, medium‐chain fatty acid, steroid, and ketone body metabolism, and positive correlations with multiple metabolites related to diacylglycerol, lysolipid, lysoplasmalogen, phospholipid, and plasmalogen metabolism. Energy balance was correlated with changes in the levels of 42 metabolites (*Q *<* *0.20; Fig. 2). Patterns included strong positive correlations with metabolites related to acyl choline metabolism, multiple inverse correlations with metabolites related to branched‐chain fatty acid, long‐chain fatty acid, and steroid metabolism, and an inverse correlation with the ketone 3‐hydroxybutyrate. Changes in metabolites were generally not correlated with energy expenditure alone, energy intake alone, or energy‐adjusted macronutrient intakes (data not shown). The sole exceptions included a strong positive correlation between energy intake and carnitine (*ρ *= 0.75, *Q *=* *0.01), and a positive correlation between energy‐adjusted protein intake and 2‐methylcitrate/homocitrate (*ρ *= 0.75, *Q *=* *0.17).

## Discussion

The major finding of this study was that a multistressor military training environment characterized by high physical activity and a short‐term but severe energy deficit elicited pronounced changes in plasma metabolite profiles that were consistent with an increase in energy metabolism, lipolysis, fatty acid oxidation, and ketogenesis. Changes in metabolites related to lipolysis and fatty acid oxidation were correlated with body mass loss and energy balance during training, whereas changes in metabolite levels were generally not correlated with energy expenditure, energy intake, or macronutrient intakes. These observations suggest that the magnitude of energy deficit induced by undereating relative to high energy expenditure, rather than macronutrient intake, may be a predominant driver of metabolic responses to military training that include severe energy deficits.

To our knowledge, only one study has adopted a metabolomics approach to capture whole‐body metabolic responses to military training. In that study, Phua et al. (2015) reported that 28 of 169 detected metabolites differentiated the urine metabolome of Singaporean soldiers measured during rest from the metabolome measured during a 6‐week combat training course during which stress, inflammation, and intestinal permeability were all increased (Li et al. 2013, 2014). Two of those 28 metabolites are known to be produced by gut bacteria, possibly reflecting changes in gut microbiota activity (Phua et al. 2015), which is supported by previously reported findings in the present cohort (Karl et al. 2017). A third discriminant metabolite, pyroglutamate, was also notable in that study because increased concentrations were associated with elevated stress, anxiety, and gastrointestinal discomfort. Pyroglutamate is a metabolite of the *γ*‐glutamyl pathway that utilizes and recovers glutathione, and can be elevated due to oxidative stress or if the *γ*‐glutamyl pathway is impaired (Lord and Bralley 2008). Although pyroglutamate was not altered in our cohort, the methionine metabolite 2‐hydroxybutyrate demonstrated the largest fold increase of any amino acid metabolite, and was one of the top compounds discriminating the post‐ and pre‐training plasma metabolomes (see Fig. 1). 2‐hydroxybutyrate, and its precursor *α*‐ketobutyrate (also increased), are formed following lysis of cystathionine in a reaction that generates cysteine, the rate‐limiting amino acid in glutathione synthesis (Lu 1998). This pathway is increased during oxidative stress, and is thought to be a marker of increased hepatic glutathione synthesis (Lord and Bralley 2008). The observed increase in 13‐hydroxyl‐octadecadienoic acid (HODE) + 9‐HODE following training is consistent with increased oxidative stress as 13‐HODE + 9‐HODE are oxidized linoleic acid metabolites that have been shown to transiently increase in plasma following exercise in association with established biomarkers of oxidative stress (Nieman et al. 2014). These observations collectively suggest that 2‐hydroxybutyrate, or other markers of glutathione metabolism, could be potential markers of oxidative stress during military training, and identify metabolic pathways impacting glutathione synthesis as potential targets for modulating oxidative stress in these environments.

The most consistent plasma metabolome signatures in the current study were within metabolic pathways related to energy metabolism. Modest elevations in several TCA cycle intermediates post‐training and lower post‐training concentrations of plasma glucose, and its glycolytic end product pyruvate indicate an overall increase in energy metabolism that was not driven by increased glycolysis. Rather, post‐training elevations in acetoacetate, 3‐hydroxybutyrate, monohydroxy fatty acids, most of the FFA and acylcarnitine forms detected, and consistent decreases in plasma diacylglycerols indicate that ketones and fatty acids were the predominant metabolic fuel after training. In support, circulating FFA derived from adipose tissue lipolysis increase in response to endurance exercise (Horowitz and Klein 2000) and energy deficit (Cahill 2006; Karl et al. 2016). Acylcarnitines facilitate transport of fatty acids into the mitochondrial matrix for *β*‐oxidation (Eaton et al. 1996) which produces monohydroxy fatty acids as intermediates, and acetyl‐CoA as an end product. In the liver, excess acetyl‐CoA can be diverted from the TCA cycle to produce the ketones acetoacetate and 3‐hydroxybutyrate. Diacylglycerols are formed during the hydrolysis of triacylglycerols to FFA and glycerol, but generally are not released into circulation (Haemmerle et al. 2002). As such, decreased concentrations likely reflect a combination of reduced dietary diacylglycerol intake, and reduced circulating triacylglycerols which has been reported following both exercise‐ and diet‐induced energy deficits (Magkos 2009).

Previous metabolomics studies have similarly demonstrated that elevated concentrations of FFA, ketones, and acylcarnitines, and in some cases, monohydroxy fatty acids comprise predominant metabolic signatures following single endurance exercise bouts (Lehmann et al. 2010; Nieman et al. 2014), brief periods of increased exercise training (Nieman et al. 2013b), and during fasting (Rubio‐Aliaga et al. 2011; Krug et al. 2012). Previous military training studies measuring targeted biomarkers of metabolic status have also reported increased circulating FFA and ketone concentrations in multistressor military training environments characterized by substantial energy deficits (Guezennec et al. 1994; Nindl et al. 1997, 2003). Our findings support those observations and demonstrate that the magnitude of increase in multiple compounds within lipid metabolite pathways were correlated with greater body mass loss and to a lesser extent energy balance, but not energy expenditure, energy intake, or macronutrient intake. Responses of acylcarnitines to energy deficit may be of particular interest as these compounds have been suggested as markers of moderate‐intensity exercise (Lehmann et al. 2010) and of the transition between anabolic and catabolic states (Krug et al. 2012), but may also have biological functions which include mediating insulin signaling, inflammation, cellular stress, and neural function (Jones et al. 2010; Schooneman et al. 2013; McCoin et al. 2015). Increased acylcarnitine concentrations have also been reported following sleep deprivation (Davies et al. 2014; van den Berg et al. 2016) suggesting that the multiple stressors experienced during military training may compound effects of energy deficit on lipid metabolism, and on the activity of pathways mediated by lipid metabolites.

Changes in plasma amino acids and associated metabolites were more variable and of lower magnitude than those observed for metabolites within lipid metabolism pathways. Increased concentrations of several BCAA catabolic intermediates such as 3‐hydroxy‐2‐ethylpropionate following training may reflect utilization of BCAA by skeletal muscle for glucogenic and ketogenic substrates (Tarnopolsky 2004; Carbone et al. 2012). In support, Berryman et al. (2017) recently reported elevated plasma amino acid concentrations, including BCAA, concomitant to increased protein breakdown following a 7‐day military training during which energy intake averaged 1.3 MJ/day. However, in contrast to those results, all amino acids in the present study, except isoleucine, were unchanged or decreased following training. Furthermore, several urea cycle intermediates were lower after training while plasma urea was increased. In combination with the clear increase in fatty acid mobilization and oxidation, these observations suggest that the timing of the post‐training plasma sample collection may have coincided with a transition from proteolysis to support gluconeogenesis toward protein‐sparing and ketogenesis to meet energy demands.

### Conclusion and perspective

In the present study, a military training exercise characterized by high energy expenditures and severe energy deficit elicited pronounced changes in plasma metabolite profiles that were consistent with an increase in energy metabolism, lipolysis, fatty acid oxidation, and ketogenesis. The exploratory analytic approach also identified markers of glutathione metabolism as potential markers of oxidative stress during military training, suggesting that metabolic pathways impacting glutathione synthesis may provide targets for modulating oxidative stress during similar training events. Changes in metabolites related to lipolysis and fatty acid oxidation were correlated with body mass loss and energy balance during training, whereas changes in metabolite levels were generally not correlated with energy expenditure, energy intake, or macronutrient intakes. As such, the magnitude of energy deficit rather than macronutrient intake appeared to be the predominant driver of metabolic responses. However, the correlative nature of the data, small sample size, and substantial overlap of intakes across groups preclude more definitive conclusions. An additional limitation is differences in the timing of post‐training sample collection, and generalizability is limited because only males of similar age were studied. Nonetheless, the study conceptually demonstrates the utility of metabolomics for advancing understanding of factors impacting physiologic responses of military personnel in austere environments.

Recent advances in metabolomics have increased accessibility and reduced costs of the analysis, allowing for a time‐ and cost‐efficient assessment of hundreds to thousands of metabolites reflecting interactions between a person's genome, transcriptome, proteome, and the environment from a single biological sample (Nicholson et al. 1999; Claus and Swann 2013; Sampson et al. 2014). The low sample volumes required and the amount of information obtained make these approaches attractive for efficiently advancing understanding of the role of nutrition in modulating metabolic responses to military training, identifying novel relationships between nutrition, metabolism, and performance decrements in these environments, and monitoring the temporal dynamics of these relationships. Findings of the present study suggest that applying metabolomics to measuring changes in markers of lipolysis, FFA oxidation, ketogenesis, and amino acid metabolism could help inform the development of strategies for monitoring metabolic status and the effectiveness of nutrition interventions during military training. Future studies conducted in these environments should examine relationships between changes in global metabolite profiles and metrics of physical and cognitive performance to help elucidate novel metabolic predictors and mediators of performance (Bradburne et al. 2015; DelRaso et al. 2015).

## Disclaimers

The opinions or assertions contained herein are the private views of the author(s) and are not to be construed as official or as reflecting the views of the Army or the Department of Defense. Citation of commercial organizations or trade names in this report does not constitute an official Department of the Army endorsement or approval of the products or services of these organizations. Opinions, interpretations, conclusions, and recommendations are those of the authors and are not necessarily endorsed by the U.S. Army.

## Conflict of Interest

No authors report a conflict of interest.

## Data Accessibility

## Supporting information




**Table S1:** Plasma metabolome before (PRE) and after (POST) a 4‐d, 51‐km cross‐country ski march.
